# Impact of larval diet on fitness outcomes of *Aedes aegypti* mosquitoes infected with *w*AlbB and *w*MelM

**DOI:** 10.1186/s13071-025-06978-7

**Published:** 2025-09-24

**Authors:** Mohd Farihan Md Yatim, Perran A. Ross, Xinyue Gu, Ary Anthony Hoffmann

**Affiliations:** Pest and Environmental Adaptation Research Group, School of BioSciences, Bio21 Institute, the University of Melbourne, Victoria, 3052 Australia

**Keywords:** *Wolbachia*, *w*MelM, *w*AlbB, Larval diet, Optimal food allocations, Fitness traits, Dengue control, *Aedes aegypti*

## Abstract

**Background:**

Releases of *Wolbachia*-infected *Aedes aegypti* are being used to effectively control diseases caused by arboviruses, such as dengue. A well-balanced larval diet is essential for producing *Wolbachia*-infected mosquitoes with optimal fitness for release.

**Methods:**

In this study, four diets with varying protein-to-carbohydrate ratios were tested with three *Ae. aegypti* lines (carrying the *w*AlbB, *w*MelM *Wolbachia* infections or uninfected) to identify optimal diets for larval rearing on the basis of diet allocations ranging from 0.4 to 3.2 mg/larva/day. The diets were selected on the basis of a review of existing literature and are characterized by progressively increasing protein and decreasing carbohydrate content: diet 1 (Pd) was based on plant-based protein (low protein and high carbohydrate), diet 2 (Kd) was based on animal-based protein (moderate protein and high carbohydrate), diet 3 (Fd) involved Hikari fish food (high protein and moderate carbohydrate), and diet 4 (IAEA) followed a widely used very-high-protein and low-carbohydrate diet developed by the International Atomic Energy Agency (IAEA). The optimal concentration for each diet was determined using a fitness index that incorporated pupation success, fecundity, hatch proportion and development time.

**Results:**

The optimal dietary allocations for diets 1–4 were 1.6, 1.2, 1.2 and 0.8 mg/larva/day, respectively, regardless of *Wolbachia* status. There was a consistent significant positive relationship between female wing length and fecundity in *w*AlbB (*r*^2^ = 0.881), *w*MelM (*r*^2^ = 0.329), and uninfected (*r*^2^ = 0.886) mosquitoes. Diet 3 (Fd) at optimal food allocation reduced a fitness cost commonly associated with the *w*AlbB line compared with the uninfected line when provided at the optimal concentration. The* w*MelM line showed a persistently low fecundity regardless of diet and concentration.

**Conclusions:**

These findings highlight the importance of an appropriate larval diet and dietary allocations in optimizing mosquito fitness for *Wolbachia*-based vector control programs. Further research into dietary composition, gut microbial interactions and *Wolbachia* associations could refine larval nutrition strategies, enhancing the effectiveness of mass-rearing for release programs.

**Graphical abstract:**

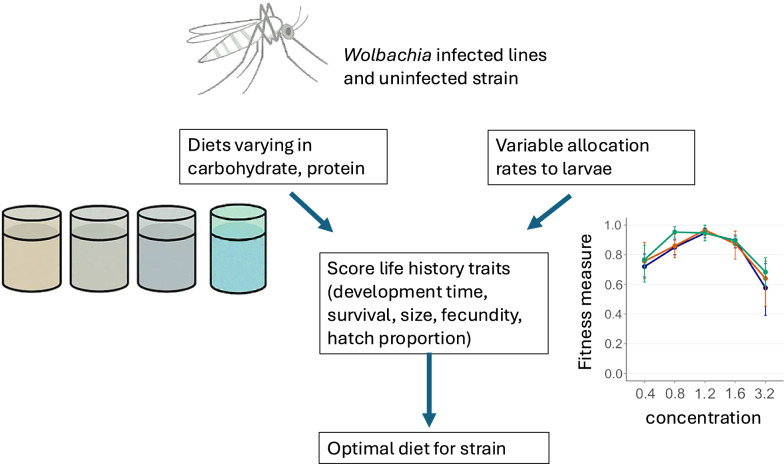

**Supplementary information:**

The online version contains supplementary material available at 10.1186/s13071-025-06978-7.

## Background

*Wolbachia* releases are being undertaken for the control of arboviruses in mosquito populations across the world [1–4]. Two general approaches have been identified: (a) the release of *Wolbachia*-infected adult males that can lead to suppression of the mosquito population through cytoplasmic incompatibility (CI), which results in non-viable offspring when infected males mate with uninfected females [5, 6], and (b) the release of both male and female *Wolbachia*-infected mosquitoes with the aim of replacing the wild mosquito population with mosquitoes carrying a *Wolbachia* strain [7–9]. These novel techniques have demonstrated success in limiting disease transmission [4, 10, 11] by controlling mosquito populations compared with conventional insecticide spraying, which carries significant environmental and economic costs [12–15]. Both *Wolbachia*-based strategies rely on the large-scale production of mosquitoes that can either compete with wild-type males for mating (suppression strategy) or establish a self-sustaining *Wolbachia*-infected population (replacement strategy) through release of females that can compete with wild females for breeding sites and transmit the *Wolbachia* infection, as well as having competitive males that induce cytoplasmic incompatibility.

Effective larval nutrition is a critical component of mosquito mass rearing programs, enabling the production of *Ae. aegypti* mosquitoes with competitive traits necessary for field success. Various diets have been tested for *Ae. aegypti* larval growth, and the most often used diets are the IAEA diet (developed by the International Atomic Energy Agency) and diets incorporating commercial fish food such as TetraMin [16, 17]. Commonly used as a standard reference in many studies, the IAEA diet consists of 50% tuna powder, 35% liver powder and 15% Brewer’s yeast [18].

A balanced protein and carbohydrate larval diet ratio can lead to the production of larger wings in *Ae. aegypti* mosquitoes [19], whereas microorganisms and algae diets result in reduced adult survival, suggesting poor nutrition [20]. In contrast, diets based on various animal-based sources (porcine, beef liver, fish food and beef liver–shrimp powder combinations) yield better development and survival [21]. However, these diets have been tested at relatively low feeding rates (average 0.41 mg/larva/day), whereas feeding rates for rearing *Aedes* mosquitoes at a medium-scale have been as high as 0.84 mg/larva/day [22]. In another study, the largest wings in *Ae. aegypti* infected with *w*Mel and *w*MelPop strain were observed when using the Tetramin fish food diet at 1 mg/larva/day [23].

Generally, most of the nutritional studies were conducted on uninfected *Ae*. *aegypti* lines. However, a study in *Drosophila* showed that *Wolbachia* can increase fertility in response to dietary changes [24]. There is a macronutrient balance that moderates the functional link between *Drosophila* and *Wolbachia*, hence affecting reproductive success [25]. In addition, under nutritional stress, *Wolbachia* may supply nutrients to the host, indicating a possible compensating function of *Wolbachia* when meal quality is poor [26]. Turning to *Ae*. *aegypti*, larval diets based on animal sources showed no negative impacts on *Wolbachia* density, development time or survival of *w*AlbB-infected *Ae*. *aegypti* [21]. However, blood meal quality, including artificial or nutrient-modified meals, was found to influence *Wolbachia*-infected mosquito fitness, especially the hatch rate [27], and larval starvation modulated *Wolbachia*'s  fitness effects [28]. These results, taken together, highlight the need of taking dietary environment and infection status into consideration while assessing mosquito fitness characteristics.

To address these issues, this study evaluates the effects of different larval diets on *Wolbachia*-infected (*w*AlbB and *w*MelM-strains described in [29, 30]) and uninfected mosquitoes by examining a range of protein and carbohydrate compositions, including both plant- and animal-based protein sources. The study examines how both diet composition and concentration affect key fitness traits, including development time, survival, wing length, fecundity and egg hatch rate across *Wolbachia*-infected and uninfected *Ae. aegypti* mosquitoes. Potential trade-offs between fitness traits are also explored, along with the influence of *Wolbachia* infection status on dietary responses. By evaluating these key life traits, we explored whether optimum diets and concentrations could help mitigate the fitness costs associated with *Wolbachia* infection in *Ae. aegypti* mosquitoes.

## Methods

### Mosquito maintenance

The *Ae. aegypti* mosquito lines used in this study were either infected with the *w*MelM or *w*AlbB strains of *Wolbachia* or were uninfected. All lines had a Cairns, Australia, genetic background and were maintained in the laboratory for at least 60 generations. The *w*MelM line is a variant of *w*Mel, derived from a field-collected *Drosophila melanogaster* and exhibits greater heat tolerance than *w*Mel [29]. The *w*AlbB line was derived from a trans-infection developed by [31] involving the transfer of the infection to *Ae. aegypti* with a Cairns, Australia, mitochondrial haplotype [30]. To ensure a consistent nuclear background, females from all three lines were backcrossed to males from a different uninfected line derived from Cairns for four generations prior to the experiment. Mosquito colonies for all diets tested were maintained under a 12-h light/12-h dark cycle at 26 °C.

### Larval diets

The composition of the diets is outlined in Table 1. These diets were selected to represent a range of protein and carbohydrate compositions. Diet 1 or the plant-based diet (Pd) contains 58% carbohydrate (C) and 17% protein (P), representing a high-carbohydrate/low-protein diet [32]; diet 2 or the Khan Diet (Kd) contains 52% (C) and 23% (P), representing a high-carbohydrate/moderate-protein diet [32, 33]; diet 3 or the Hikari fish food (Fd) diet contains 24% (C) and 36% (P), representing a moderate-carbohydrate/high-protein diet; diet 4 or the IAEA [18] diet contains 2% (C) and 72% (P), representing a low-carbohydrate/very-high-protein diet. The complete macronutrient content of all diets is presented in Table 2.

**Table 1:** Ingredients list for all four diets (10 g/diet)

Diet 1 (Pd)	Diet 2 (Kd)	Diet 3 (Fd)	Diet 4 (IAEA)
Bean (2 g)	Bean (1.2 g)	Hikari Fish Food (10 g)	Brewer’s Yeast (1.5 g)
Chickpea (2 g)	Chickpea (1.8 g)	**Ingredients**: fish meal, wheat germ meal, soybean meal, wheat flour, whole crushed silkworm pupae, dried seaweed meal, dried bakery product, brewers dried yeast, fish oil, krill meal, spirulina, garlic, DL-methionine, astaxanthin, choline chloride, vitamin E supplement, L-ascorbyl-2-polyphosphate (stabilized vitamin C), inositol, d-calcium pantothenate, riboflavin, vitamin A supplement, thiamine mononitrate, pyridoxine hydrochloride, niacin, folic acid, vitamin D3 supplement, biotin, disodium phosphate, ferrous sulfate, magnesium sulfate, zinc sulfate, manganese sulfate, copper sulfate and calcium iodate	Liver powder (3.5 g)
Mung bean (2 g)	Corn (1.8 g)		Tuna powder (5 g)
Mushroom (2 g)	Liver powder (2.2 g)		
Rice (2 g)	Rice (1.8 g)		
	Wheat (1.2 g)		
10 g	10 g	10 g	10 g

**Table 2:** Macronutrient content in all diets (per 10 g; percentage (%) also shown)

Macronutrients	Diet 1 (Pd)	Diet 2 (Kd)	Diet 3 (Fd)	Diet 4 (IAEA)
Carbohydrate	5.758 g	5.151 g	2.42 g	0.16 g
	(57.58%)	(51.51%)	(24.20%)	(1.6%)
Protein	1.698 g	2.348 g	3.6 g	7.17 g
	(16.98%)	(23.48%)	(36.00%)	(71.70%)
Fat	0.3 g	0.520 g	0.9 g	1.115 g
	(3.00%)	(5.20%)	(9.00%)	(11.15%)

### Ingredient preparation

Commercially available wheat flour (White Wings Premium Plain Flour; White Wings, Australia), bean (White bean, Phaseolus vulgaris, McKenzie’s Foods, Australia), rice flour (Erawan Brand; Cho Heng Rice Vermicelli Factory Co. Ltd., Thailand), corn flour (Sunflower Corn Flour; Singapore), chickpea flour (McKenzie’s Australian Chick Pea Flour; McKenzie’s Foods, Australia), mung bean flour (Xinliang Mung Bean Starch; China), beef liver powder (Barbell Organic Beef Liver; Barbell Foods, Australia), Brewer’s yeast (Macro Wholefoods Market; Woolworths, Australia) and Hikari fish food (Hikari Tropical Sinking Wafers; Kyorin Food Industries, Japan) were used. Fresh mushrooms (*Agaricus bisporus*) and tuna in water (Ocean Rise®; ALDI Stores, Australia) were chopped into small pieces, dried at 50 °C for 16 h, and ground into powder. All flours and powders were sifted through a fine strainer, and a 2% solution was prepared by mixing 10 g of each diet with 500 mL of reverse osmosis (RO) water. To maintain the differences in food allocation, different volumes (1–8 mL) were added to a container each day depending on the concentration of food being maintained. This did result in some variation in the total volume in a container by the end of the experiment per diet (up to 63 mL when the most extreme allocations are considered but usually much less than this).

### Larval development time and survival (pupation success)

Eggs from each mosquito line were hatched in reverse osmosis (RO) water with a small amount of Brewer’s yeast. After 12 h, 50 first-instar larvae were transferred into plastic containers 170 mm (length) × 119 mm (width) × 60 mm (height) filled with 500 mL of RO water and assigned to diet treatments at concentrations of 0.4, 0.8, 1.2, 1.6 and 3.2 mg/larva/day. Note that we use the term ‘concentration’ and ‘allocation’ interchangeably in the paper because a higher allocation is expected to result in a higher concentration of food. Each concentration/diet/line combination was tested as six replicates (300 larvae in total per treatment). Across the three mosquito lines, 4500 larvae were used per diet, resulting in a total of 18,000 larvae used for all four diets. Because our initial interest was to compare strains across a range of concentrations/food allocations to define optimal conditions, the size of the experiments precluded testing all diet treatments at the same time in a randomized design and thus different mosquito generations were used for each diet treatment. Specifically, diets 1, 2, 3 and 4 were tested in separate months using generations F5, F6, F7 and F8, respectively. Nevertheless, all experiments were conducted under the same conditions outlined above in terms of temperature, humidity and rearing protocols.

Larval development time, defined as the duration from egg hatching to pupation, was recorded twice daily (morning and evening) starting from the onset of pupation. Pupae were counted, sex was determined and later pupae were transferred to designated containers on the basis of their respective lines and concentrations. This process was followed for all diet treatments. Survival was defined as the proportion of first-instar larvae that successfully pupated, that is the number of pupae divided by the initial number of first-instar larvae.

### Wing length measurement

A total of 15 2- to 4-day-old adult males and females from each concentration and line were selected haphazardly and preserved in 100% ethanol for wing length measurement. Wings were separated using forceps under a stereomicroscope. Measurements were taken using a Nikon SMZ1500 microscope equipped with a camera and IS Capture software. Wing length was determined as the distance from the alular notch to the radius 3 vein.

### Fecundity and egg hatch rates

All concentration/line combinations for a diet were blood fed on the same day; however, owing to differences in larval development time across diet concentrations, the age of females at the time of blood feeding varied between 4- and 7-days post-emergence. After blood feeding, 20 fully engorged females were transferred to small plastic containers lined with sandpaper (*Norton Master Painters P80*; Saint-Gobain Abrasives Pty. Ltd., Thomastown, Victoria, Australia) and partially filled with RO water to facilitate oviposition.

Eggs were collected by removing the sandpaper after 4 days. Females that did not lay eggs within this period remained in the containers until day 7 to account for potential late oviposition. Collected eggs were stored in plastic containers lined with tissue paper to prevent excess moisture. The eggs were kept at 26 °C for 3 days before hatching. On the third day, RO water and a small amount of Brewer’s yeast (*Macro Wholefoods Market*; Woolworths, Australia) were added to the container to stimulate hatching. After 24 h, fecundity was measured as the total number of eggs per female, while hatch proportion was calculated as the total number of hatched eggs divided by the total number of eggs laid.

### Statistical analysis

Both generalized linear models and analyses of variance (ANOVAs) were used to analyse trait data depending on the distribution of trait data and associations between trait means and variances (Supplementary information) as assessed through Levene’s tests. The main analyses focussed on line and concentration effects within diets. For development time, generalized linear models with a gamma distribution and log link were applied, while survival and hatch proportions were analysed using generalized linear models with a Poisson distribution assumed. For fecundity where there was a negative association between line means and variances, generalized linear models with a negative binomial distribution were applied. A few females that failed to produce any eggs were excluded from this analysis. All generalized linear models were fitted using a covariance matrix with robust estimators. For wing length (male and female) traits which tended to be normally distributed, two-way ANOVAs were performed to assess mosquito lines and diet concentrations/food allocations. Partial Eta squared values (η^2^) were computed to reflect effect sizes. As each diet was tested separately, our initial analyses did not involve a direct comparison across diets. However, to get some idea about the relative performance of the lines across the diets, line performance was compared at the optimal concentration/food allocation using the above mentioned models and analyses but with a focus on diet and line as factors. Pairwise comparisons across lines within each diet are also presented as part of these analyses, with the Bonferroni correction applied where generalized linear models were used, and Tukey B post hoc tests being applied where ANOVAs were carried out. Pearson correlation analysis was conducted to assess the relationship between female wing length and fecundity.

The optimal allocation/concentration for each diet was determined by computing life table scores through multiplying the averages of fecundity, hatch proportion, and pupation success (survival) proportion, and then dividing by the development time [34]. All statistical analyses were performed using IBM SPSS Statistics version 29. Graphs were generated using RStudio (version 2024.12.0, Build 467) or IBM SPSS Statistics version 29, and further graphical edits were performed in Inkscape (version 1.4). Means are presented in graphs with bootstrap 95% confidence intervals (CI).

## Results

### Development time

**Fig. 1:**
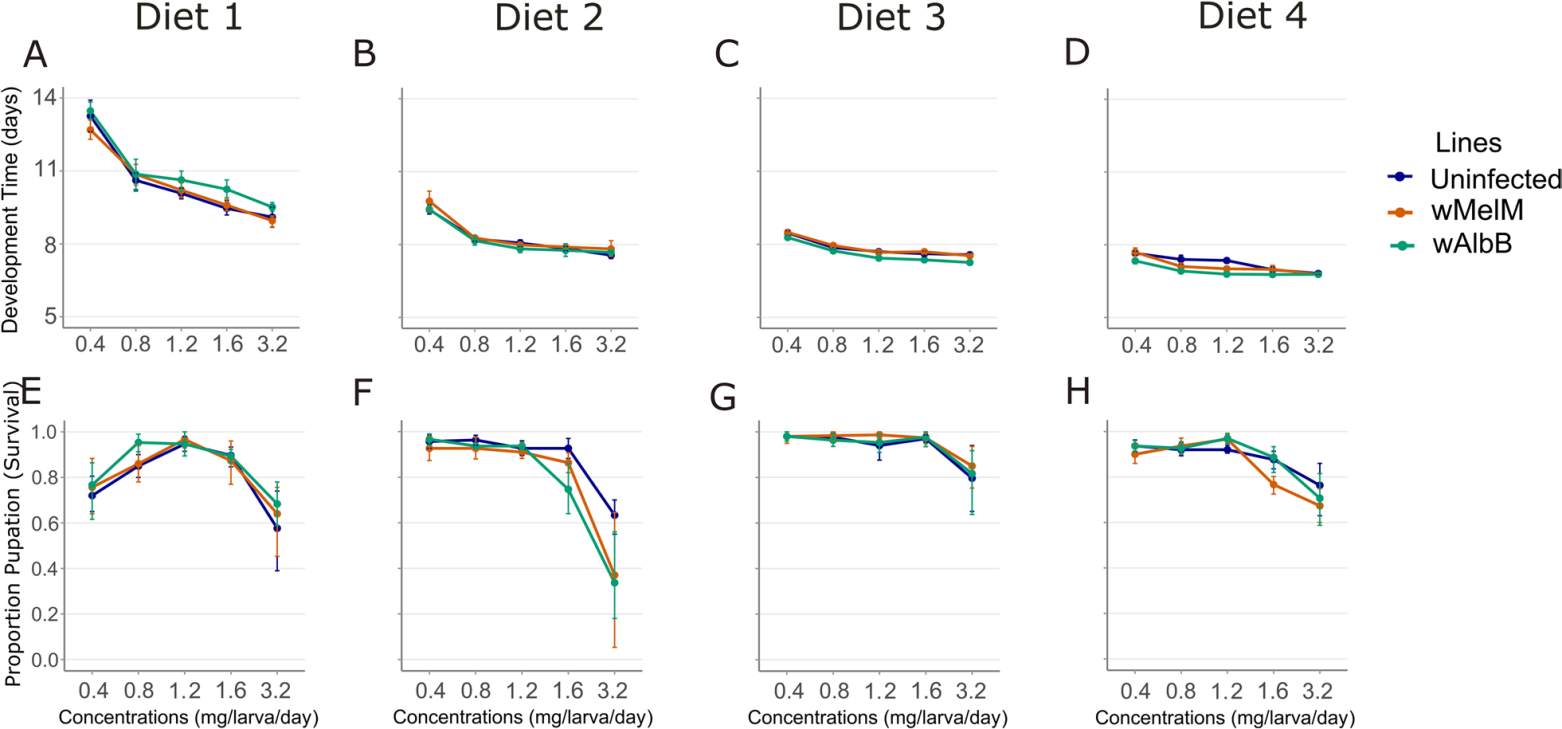
Mean development time (**A**–**D**) and proportion of pupation success (survival) (**E**–**H**) of *Aedes aegypti* across four larval diets (diets 1–4) with varying protein-to-carbohydrate ratios and concentrations (0.4–3.2 mg/larva/day). Data are presented for three mosquito lines: uninfected (blue), *w*MelM (orange), and *w*AlbB (green). Error bars represent 95% bootstrapped confidence intervals (CI)

The generalized linear model analyses showed a significant effect of lines and concentration on mosquito development time across all diets (Fig. 1A–D; Tables 3–6; all *P* < 0.016). Higher allocations consistently resulted in shorter development times, indicating a strong dose-dependent relationship (Fig. 1A–D). Significant interaction effects between mosquito lines and concentration were observed for diet 3 (Fd) and diet 4 (IAEA) (Tables 5, 6; *P* < 0.016), suggesting variability in how mosquito lines responded to changes in food concentration in these diets.

**Table 3:** Summary of statistical results for mosquito traits under diet 1 (Pd) on the basis of generalized linear models for development time, survival proportion, fecundity and hatch proportion, and on two-way analyses of variance (ANOVAs) for wing length (male and female)

Dependent variable	Source	df	Mean square/effect size (*η*^*2*^*)*	*F/*Wald Chi Square	*P*-value
Development time	Line	2		21.460	< 0.001
	Concentration	4		818.977	< 0.001
	Line × concentration	8		7.692	0.464
Survival proportion	Line	2		3.992	0.136
	Concentration	4		102.544	< 0.001
	Line × concentration	8		7.648	0.469
Wing length (male)	Line	2	0.028/0.031	3.316	0.038
	Concentration	4	0.492/0.522	57.246	< 0.001
	Line × concentration	8	0.016/0.065	1.816	0.076
	Error	210	0.009		
Wing length (female)	Line	2	0.024/0.023	2.456	0.088
	Concentration	4	1.108/0.681	112.250	< 0.001
	Line × concentration	8	0.042/0.140	4.280	< 0.001
	Error	210	0.010		
Fecundity	Line	2		37.219	< 0.001
	Concentration	4		34.335	< 0.001
	Line × concentration	8		14.109	0.079
Hatch proportion	Line	2		58.708	< 0.001
	Concentration	4		30.001	< 0.001
	Line × concentration	8		38.350	< 0.001

**Table 4:** Summary of statistical results for mosquito traits under diet 2 (Kd) based on generalized linear models for development time, survival proportion, fecundity and hatch proportion, and on two-way ANOVAs for wing length (male and female)

Dependent variable	Source	df	Mean square/effect size (*η*^*2*^*)*	F/Wald Chi Square	*P*-value
Development time	Line	2		8.305	0.016
	Concentration	4		603.102	< 0.001
	Line × concentration	8		10.652	0.222
Survival proportion	Line	2		11.630	0.003
	Concentration	4		352.904	< 0.001
	Line × concentration	8		17.919	0.022
Wing length (male)	Line	2	0.022/0.031	3.399	0.035
	Concentration	4	0.239/0.412	36.736	< 0.001
	Line × concentration	8	0.022/0.116	3.445	< 0.001
	Error	210	0.006		
Wing length (female)	Line	2	0.331/0.192	24.983	< 0.001
	Concentration	4	0.662/0.488	49.965	< 0.001
	Line × concentration	8	0.072/0.171	5.410	< 0.001
	Error	210	0.013		
Fecundity	Line	2		82.029	< 0.001
	Concentration	4		130.040	< 0.001
	Line × concentration	8		47.694	< 0.001
Hatch proportion	Line	2		4.760	0.093
	Concentration	4		9.021	0.061
	Line × concentration	8		8.147	0.419

**Table 5:** Summary of statistical results for mosquito traits under diet 3 (Fd) based on generalized linear models for development time, survival proportion, fecundity and hatch proportion, and on two-way ANOVAs for wing length (male and female)

Dependent variable	Source	df	Mean square/effect size (*η*^*2*^*)*	*F*/Wald Chi Square	*P*-value
Development time	Line	2		171.535	< 0.001
	Concentration	4		950.553	< 0.001
	Line × concentration	8		18.874	0.016
Survival	Line	2		2.733	0.255
	Concentration	4		61.617	< 0.001
	Line × concentration	8		4.138	0.844
Wing length (male)	Line	2	0.003/0.007	0.781	0.459
	Concentration	4	0.209/0.489	50.278	< 0.001
	Line × concentration	8	0.013/0.104	3.047	0.003
	Error	210	0.004		
Wing length (female)	Line	2	0.013/0.013	1.365	0.258
	Concentration	4	0.768/0.599	78.412	< 0.001
	Line × concentration	8	0.018/0.066	1.849	0.070
	Error	210	0.01		
Fecundity	Line	2		82.047	< 0.001
	Concentration	4		25.893	< 0.001
	Line × concentration	8		8.616	0.376
Hatch proportion	Line	2		36.215	< 0.001
	Concentration	4		8.184	0.085
	Line × concentration	8		36.420	< 0.001

**Table 6:** Summary of statistical results for mosquito traits under diet 4 (IAEA) based on generalized linear models for development time, survival proportion, fecundity and hatch proportion, and on two-way ANOVAs for wing length (male and female)

Dependent variable	Source	df	Mean square/effect size (*η*^*2*^*)*	*F*/Wald Chi Square	*P*-value
Development time	Line	2		134.717	< 0.001
	Concentration	4		471.118	< 0.001
	Line × concentration	8		68.129	< 0.001
Survival proportion	Line	2		3.583	0.167
	Concentration	4		158.606	< 0.001
	Line × concentration	8		31.151	< 0.001
Wing length (male)	Line	2	0.043/0.092	10.644	< 0.001
	Concentration	4	0.011/0.049	2.725	0.030
	Line × concentration	8	0.005/0.045	1.232	0.282
	Error	210	0.004		
Wing length (female)	Line	2	0.147/0.118	14.105	< 0.001
	Concentration	4	0.036/0.062	3.462	0.009
	Line × concentration	8	0.020/0.068	1.909	0.060
	Error	210	0.010		
Fecundity	Line	2		51.559	< 0.001
	Concentration	4		10.918	0.027
	Line × concentration	8		18.735	0.016
Hatch proportion	Line	2		37.313	< 0.001
	Concentration	4		3.095	0.542
	Line × concentration	8		18.828	0.016

### Survival proportion (pupation success)

Concentration significantly influenced the proportion of larvae surviving to the pupal stage across all diets (Fig. 1E–H; Tables 3–6; all *P* < 0.001). Mosquito line effects were only significant for diet 2 (Kd) (Table 4; *P* = 0.003). Interaction effects between mosquito lines and concentration were not significant for diet 1 (Pd) and diet 3 (Fd). However for diet 2 (Kd) and diet 4 (IAEA) interaction effects were significant (Tables 4 and 6; *P* < 0.022).

### Male wing length

**Fig. 2:**
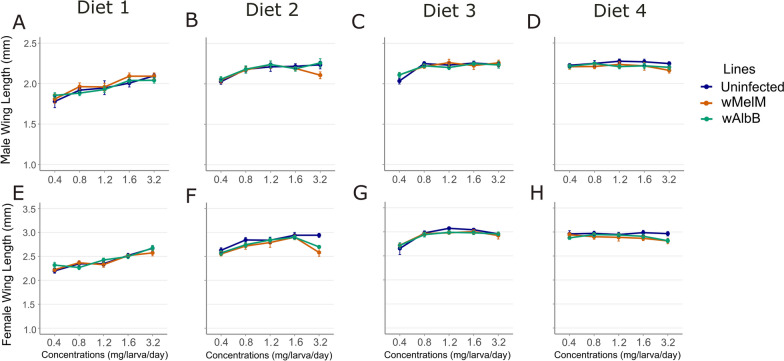
Mean of male (**A**–**D**) and female (**E**–**H**) wing lengths of *Aedes aegypti* reared on four larval diets (diets 1–4) across five concentrations (0.4–3.2 mg/larva/day). Results are shown for uninfected (blue), *w*MelM (orange), and *w*AlbB (green) lines. Error bars represent 95% bootstrapped confidence intervals (CI)

Two-way ANOVAs showed that concentration significantly influenced male wing length in all diets (Fig. 2A–D; Tables 3–6; all *P* < 0.030). Mosquito line significantly affected male wing length in diets 1 (Pd), 2 (Kd), and 4 (IAEA) (*P* < 0.038), but not in diet 3 (Fd) (*P* = 0.459). Significant interaction effects between mosquito line and concentration were observed in diet 2 (Kd) (Table 4; *P* < 0.001) and diet 3 (Fd) (Table 5; *P* = 0.003), indicating that the impact of concentration varied by mosquito line for these diets.

Increasing food concentrations significantly enhanced male wing length, with the strongest effects observed for diets 1 (Pd)–3 (Fd) (Fig. 2A–D; Tables 3–5; all *P* < 0.001). For diet 4 (IAEA), the relationship was weaker, but the concentration effect was still statistically significant (*P* = 0.030). Differences between mosquito lines were generally smaller, varying by diet and concentration and indicating no consistent pattern of line superiority across all diets.

### Female wing length

In two-way ANOVAs, food concentration significantly influenced female wing length across all diets (Fig. 2E–H; Tables 3–6; all *P* < 0.009). Mosquito line had a significant effect on female wing length only in diets 2 (Kd) and 4 (IAEA) (Fig. 2F, H; Tables 4 and 6; both *P* < 0.001). In addition, significant interaction effects between mosquito line and concentration were found in diets 1 (Pd) and 2 (Kd) (Fig. 2E, F; Tables 3 and 4; both *P* < 0.001), suggesting that the effect of food concentration on wing length varied by mosquito line in these diets.

### Fecundity

**Fig. 3:**
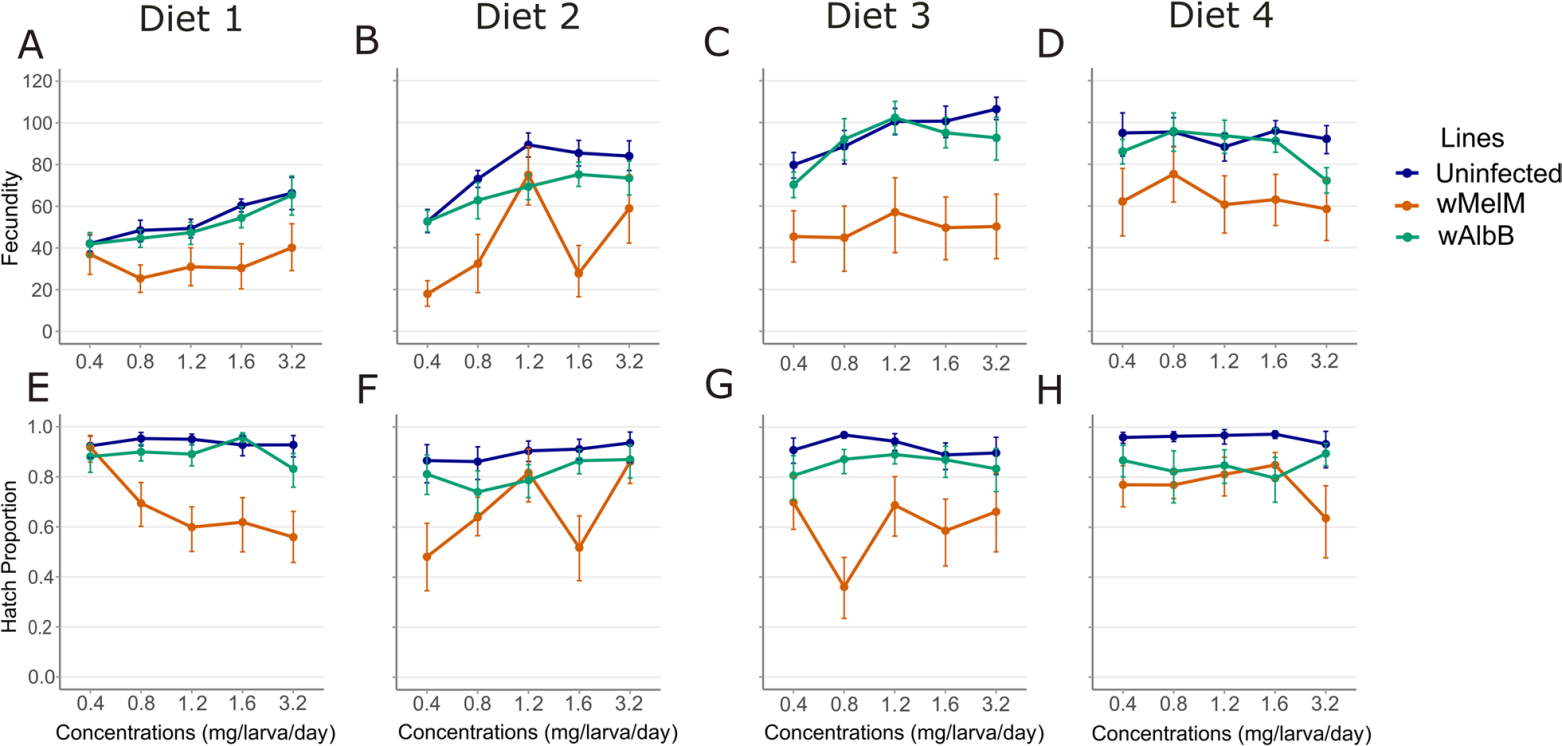
Mean fecundity (**A**–**D**) and hatch proportion (**E**–**H**) of *Aedes aegypti* females reared on four larval diets (diets 1–4) across five concentrations (0.4–3.2 mg/larva/day). Data are shown for uninfected (blue), *w*MelM (orange), and *w*AlbB (green) mosquito lines. Error bars represent 95% bootstrapped confidence intervals (CI)

The generalized linear model with negative binomial distribution indicated significant effects of mosquito lines and concentration on fecundity across all diets (Fig. 3A–D; Tables 3–6, *P* < 0.027). Significant interactions between mosquito lines and concentration were observed only in diet 2 (Kd) and diet 4 (IAEA) (Tables 4 and 6; *P* < 0.016), reflecting an effect of concentration on fecundity differences among mosquito lines in these diets.

The uninfected line typically exhibited higher fecundity compared with the *w*MelM and *w*AlbB lines (Fig. 3A–D). In addition, *w*MelM consistently had the lowest fecundity, significantly differing from both uninfected and *w*AlbB lines across all diets with a larger spread of fecundity scores.

### Hatch proportion

The generalized linear models indicated significant differences in hatch proportion among mosquito lines across diets (Fig. 3E–H; Table 3, 5–6; diets 1, 3 and 4; all *P* < 0.001). Concentration significantly influenced hatch proportion only in diet 1 (Pd) (*P* < 0.001). Interaction effects between mosquito line and concentration were significant for diets 1 (Pd), 3 (Kd) and 4 (IAEA) (Table 3, 5 and 6; all *P* < 0.016).

### Relationship between female wing length and fecundity

**Fig. 4:**
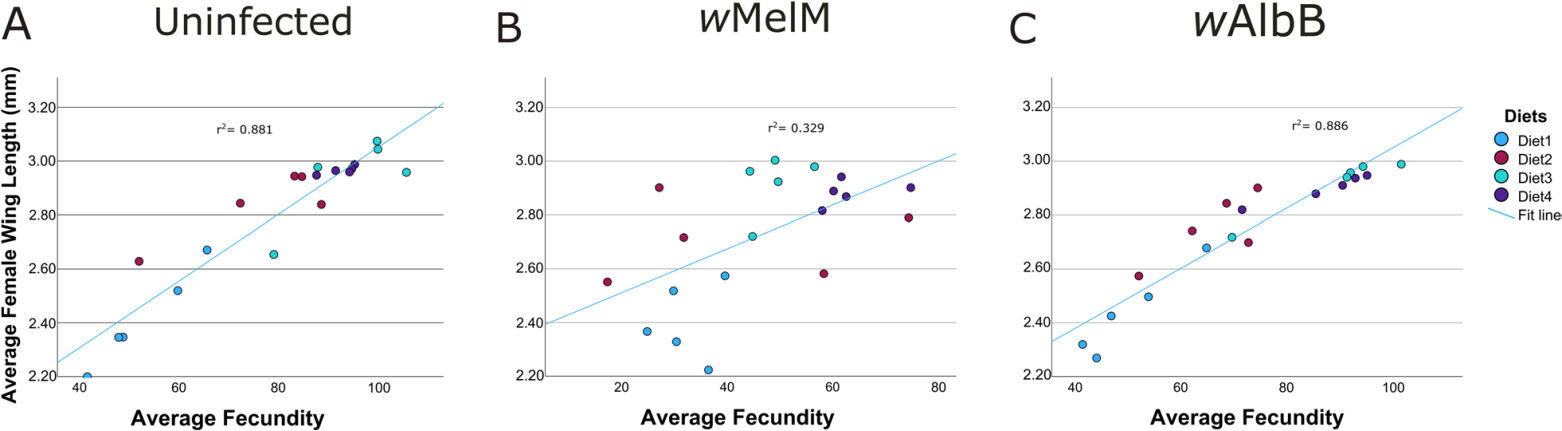
Relationship between mean female wing length and mean fecundity in uninfected (**A**), *w*MelM (**B**), and *w*AlbB (**C**) *Aedes aegypti* lines across all diets and concentrations/allocations. Each point represents the mean values per diet treatment across all allocations; colours indicate the corresponding diet

For female mosquitoes, a strong positive correlation was observed between wing length and fecundity (Fig. 4) in both the uninfected (*r*^2^ = 0.881) and *w*AlbB (*r*^2^ = 0.886) lines when combining data across diets, indicating that larger females consistently produced more eggs. The relationship was mostly linear except at higher fecundities, and the data suggest that wing length is a reliable predictor of fecundity in these two lines. In *w*MelM, there was a weaker association (*r*^2^ = 0.329). The reasons for this were unclear since the fecundity and wing length values for this line covered a similar or greater range of values than for the other lines).

### Optimal concentration

**Fig. 5:**
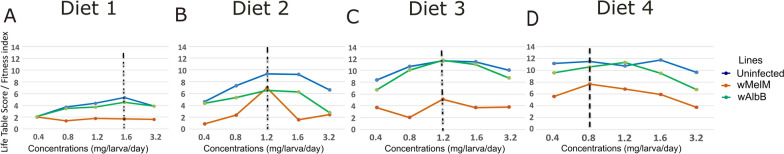
Fitness index (life table score) across different diet concentrations for uninfected, *w*MelM, and *w*AlbB *Aedes aegypti* lines reared on four larval diets (**A**–**D**). The fitness index was calculated using life table parameters, following the method proposed by [34]. Each plot shows the performance of the three mosquito lines across five concentrations (0.4–3.2 mg/larva/day), with the vertical dashed line indicating the concentration yielding the highest fitness index (optimal concentration)

The fitness index used in this study was developed on the basis of mosquito fitness traits, following the method described elsewhere [34]. The optimal concentration was 1.6 mg/larva/day for diet 1 (Pd) and 1.2 mg/larva/day for diet 2 (Kd) and diet 3 (Fd). For diet 4 (IAEA), the optimum concentration was 0.8 mg/larva/day, indicating that diet composition and concentration influence overall mosquito fitness (Fig. 5). Almost all mosquito lines reached their maximum fitness index at identical concentrations within each diet, suggesting that the quantity of food at a specific composition is more influential in determining fitness than features unique to each line. The similar patterns across lines may indicate a common pattern of nutrient absorption that optimizes performance for each dietary type.

**Fig. 6:**
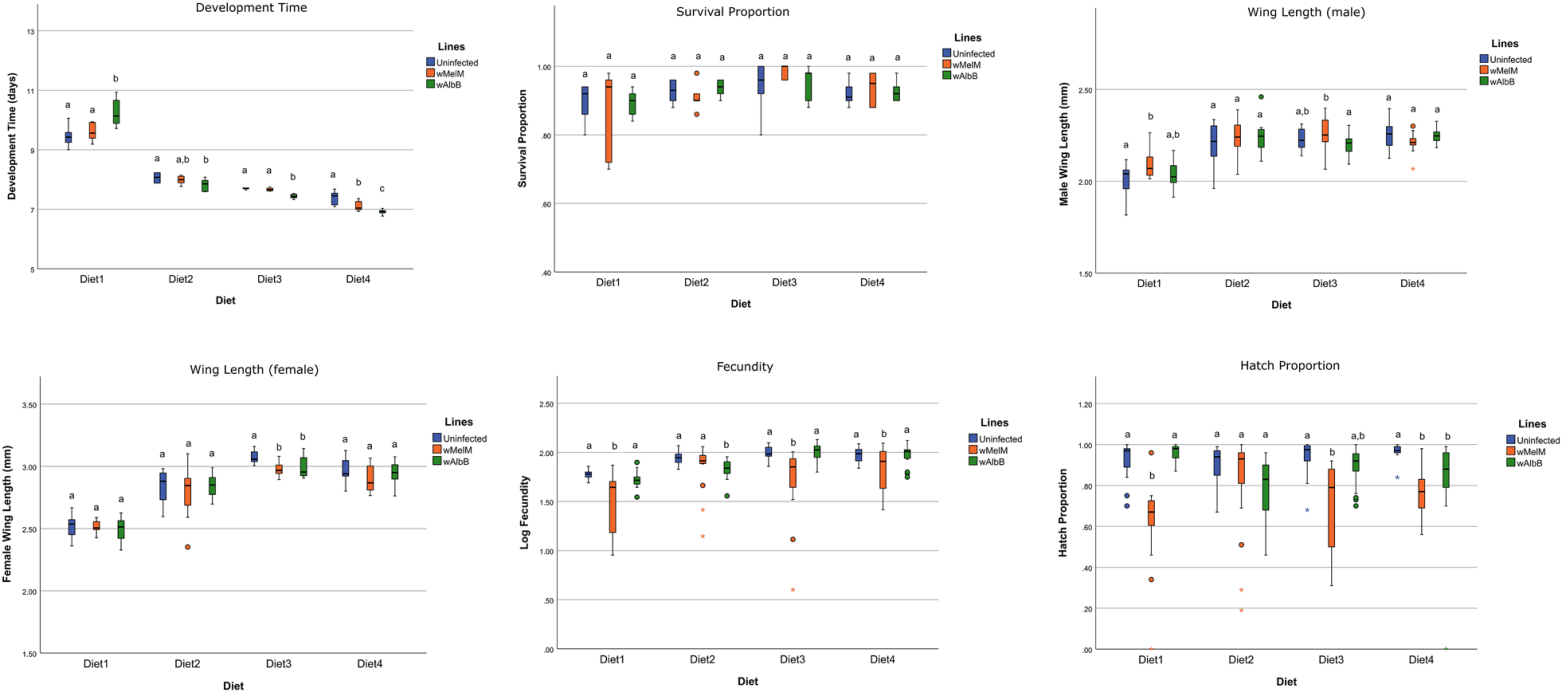
Comparison of diets at optimal concentrations for the fitness traits. Different letters indicate significant differences in pairwise comparisons within each diet between mosquito lines, based on Tukey B post hoc tests (for wing length measures) or Bonferroni-corrected comparisons (for other traits). In the boxplot, asterisks (^*^) indicate extreme outliers (greater than 3 times the interquartile range), while circles (O) represent moderate outliers (between 1.5 times and 3 times the interquartile range)

To compare diets directly, we ran comparisons of diets at optimal concentrations on the fitness traits (Table 7). However, this should be interpreted with caution, as each experiment was conducted separately. At optimal concentrations, main effects of line were observed for female wing length, fecundity and hatch proportion (all *P* < 0.014). Main effects of diets were evident for all traits (all *P* < 0.001). Development time was shortest for diet 4 (IAEA), survival was highest for diet 3 (Fd), and wing length and fecundity tended to be lower for diet 1 (Pd); hatch proportion showed an interaction effect with *w*MelM performing similar to the other lines on diet 2 (Kd) but not the other diets (Fig. 6).

**Table 7:** Summary of statistical analyses of mosquito traits when measured at optimum concentrations of each diet based on generalized linear models for development time, survival proportion, fecundity and hatch proportion, and on two-way ANOVAs for wing length (male and female)

Dependent variable	Source	df	Mean square/effect size (*η*^*2*^*)*	*F*/Wald Chi Square	*P*-value
Development time	Line	2		3.673	0.159
	Diet	3		1093.498	< 0.001
	Diet × line	6		36.535	< 0.001
Survival proportion	Line	2		1.975	0.373
	Diet	3		15.564	0.001
	Diet × line	6		7.273	0.296
	Error				
Wing length (male)	Line	2	0.011/0.022	1.920	0.150
	Diet	3	0.390/0.544	66.761	< 0.001
	Diet × line	6	0.014/0.077	2.323	0.035
	Error	168	0.006		
Wing length (female)	Line	2	0.044/0.049	4.373	0.014
	Diet	3	2.205/0.798	221.547	< 0.001
	Diet × line	6	0.010/0.034	0.989	0.434
	Error	168	0.10		
Fecundity	Line	2		35.991	< 0.001
	Diet	3		92.028	< 0.001
	Diet × line	6		28.046	< 0.001
Hatch proportion	Line	2		32.785	< 0.001
	Diet	3		23.049	< 0.001
	Diet × line	6		30.748	< 0.001

## Discussion

A possible limitation of this study is the minor variation in larval rearing water volume across treatments, particularly at the lowest and highest feeding levels. This variation was due to differences in the volume of food slurry required to deliver accurate and homogeneous feeding doses. The primary objective of the study was to determine the optimal food allocation for each diet, and the wide feeding range was chosen to capture both the lower and upper thresholds for larval survival. To minimize larval crowding effects, the initial rearing water was standardized at 500 mL per 50 larvae (1 larva: 10 mL). However, achieving homogenous slurry concentrations above 4% proved challenging, especially for complex diets with multiple ingredients. Therefore, to ensure consistency in food delivery across treatments, feeding volumes and consequently, the amount of total rearing water differed (by a maximum of 12.6%). While this introduces a confounding factor, it was necessary to maintain slurry homogeneity. Similar approaches have been adopted in previous studies where feeding volume adjustments led to variation in rearing water volume [35, 36].

Diet allocation significantly influenced most mosquito fitness traits, as shown in Tables 3–6 and Figs. 1–3. In particular, traits such as development time, survival and wing length showed strong responses to changes in allocation. The fitness traits often tended to increase at intermediate concentrations before decreasing again at high concentrations of larval food. Effects of line (*w*MelM, *w*AlbB or uninfected) were also detected, with uninfected larvae tending to perform better in fecundity and egg hatch rate, as shown in Fig. 3.

The overall impact of line was less evident compared with diet concentrations. However, for the *w*MelM line, reduced fecundity consistently occurred across all diets. This reduction was probably related to blood meal rather than to larval diet. A low fecundity for a *w*Mel *Wolbachia* infection related to *w*MelM was also reported in another study [37]. However, for diet 2 (Kd) at 1.2 mg/larva/day concentration, the *w*MelM line showed a relatively high fecundity and hatch proportion. The reason for this abrupt change is unclear and is perhaps related to an uncontrolled lower *Wolbachia* density in this treatment. Further analysis of *Wolbachia* density of the *w*MelM line across food allocations will help to test this hypothesis.

In high-carbohydrate diets, the protein composition of the larval diet strongly influenced mosquito fitness traits. Increased protein content significantly improved developmental outcomes, supporting previous findings on the critical role of dietary protein in larval growth, development rate and adult size in *Ae. aegypti* and related species [38–40]. Under a plant-based, low-protein diet, the proportion of pupation success remained relatively high despite delayed development, indicating that, while energy availability can sustain survival, it is insufficient for optimal growth. In other work, replacing mushroom powder with animal-based liver powder markedly enhanced fitness traits and reduced development time [32].

When comparing a balanced diet (diet 3 (Fd)) with a very high-protein, low-carbohydrate diet (diet 4 (IAEA)), the fastest larval development was observed under diet 4 (IAEA) across all *Ae. aegypti* lines. This is consistent with earlier findings showing that elevated dietary protein levels enhance developmental speed [41, 42]. However, this rapid development was accompanied by reduced female wing length, suggesting an environmentally based trade-off between development speed and adult fitness. A more balanced protein-to-carbohydrate ratio larval diet (LRD) produced larger females in uninfected *Ae. aegypti* compared with a high-protein, low-carbohydrate diet [41]. In contrast, male wing length showed similar trends across both diet 3 (Fd) and diet 4 (IAEA), with minimal variation between diets or lines. This suggests a potential physiological constraint on male body size, whereby males may reach a species–specific upper limit regardless of nutritional conditions. This observation aligns with previous findings showing that female size was more responsive to dietary variation, while male size remained relatively stable across nutritional conditions [19]. Here, we only considered morphological measurements linked to mosquito size, and it would also be worth investigating dietary effects on other more general measures of size such as the dry mass of adults, which might link to other aspects of male fitness, and the wet weight of females following emergence and blood feeding. Larger females tend to have higher fecundity, as larger individuals have a better ability to hold a greater volume of blood and possess higher energy reserves for egg production [43]. A diet with a balanced carbohydrate and protein content tends to result in higher lipid reserves at the adult stage, compared with a high-protein, low-carbohydrate diet, which can lead to reduced size and lower energy storage despite faster development [44].

Diet 3 (Fd) and diet 4 (IAEA) consistently yielded higher fitness index scores in *Ae. aegypti* than the carbohydrate-rich, low-protein diet. Among the diets tested, diet 3 (Fd), supported the highest composite life table scores across in *w*AlbB and uninfected line at optimal concentration for maximizing development, survival and reproductive output. These results are consistent with the possibility that improved larval nutrition could mitigate *Wolbachia*-associated fitness costs [7, 45] particularly for *w*AlbB-infected mosquitoes, which in this study, performed nearly as well as the uninfected line at optimum concentration. The narrowing gap in life table scores between *w*AlbB and uninfected mosquitoes suggests that nutritional quality can buffer fitness costs and improve competitiveness. In *w*MelM, reduced fecundity was observed despite limited changes in wing length, suggesting that other contributing factors beyond larval nutrition may be present. This is consistent with predictions from metabolic modelling studies indicating that *Wolbachia* strains such as *w*Mel may rely on host-derived lipids and amino acids for growth, potentially influencing pathways relevant to reproduction. [46]. Blood source may also be a factor since a previous study on *w*MelM using diet 3 (Fd) but with a different human volunteer did not identify any costs to fertility [29].

Although microbiome measurement was not performed in this study, several studies have found that the microbiome in the mosquito gut is essential for digestion, immune function and high mosquito fitness [47–50]. High larval food allocation (up to 2 mg/larva/day) can increase gut microbiome diversity, which can remain at a high level even after eclosion and blood feeding [51]. We found that mosquito fitness traits tended to be maximized at a food allocation of 0.8–1.2 mg/larva/day, depending on the diet. This raises an interesting question as to whether these intermediate levels of food availability influence microbiome diversity and composition in ways to potentially enhance mosquito fitness.

## Conclusions

In this study, we found that diet composition and allocation play a significant role in determining key life traits of mosquitoes. A well-balanced protein and carbohydrate content produces mosquitoes with higher fitness compared with diets with lower protein content. The uninfected mosquito line showed the highest overall fitness across all diets compared with the *w*MelM and *w*AlbB lines. However, with a balanced diet and at an optimum concentration, the *w*AlbB line seems to mitigate the fitness cost of *Wolbachia* infection. In contrast, the *w*MelM line showed lower fecundity across most diets, probably owing to factors other than the larval diet. Overall, almost all mosquito lines achieved maximum fitness scores at the same concentration. Diet 3 at 1.2 mg/larva/day yielded the highest performance on the basis of the fitness index, and it most effectively minimized the impact of *Wolbachia* infection, particularly in the *w*AlbB line. However, the different responses among mosquito lines also suggest that other factors such as gut microbiota could play an important role in mediating nutrient utilization and overall fitness. Further microbiome profiling using 16S rRNA gene sequencing could improve our understanding of these interactions. This, in turn, could enhance *Wolbachia* release strategies to control arboviral infections. Such approaches would greatly benefit the consistency, scalability and success of mass-release programs aiming to suppress or replace disease-transmitting mosquito populations.

## Supplementary information

Additional file 1.

## Data Availability

The datasets analysed during the current study are available on Figshare at: 10.26188/28785869.
