# Supplementary material for: Impact of larval diet on fitness outcomes of Aedes aegypti mosquitoes infected with wAlbB and wMelM
Source: Parasit Vectors. 2025 Sep 24;18:386. doi: 10.1186/s13071-025-06978-7 (PMC12461977; doi:10.1186/s13071-025-06978-7)

**Supplementary information**

Distribution of traits across the different diets collated across concentrations.


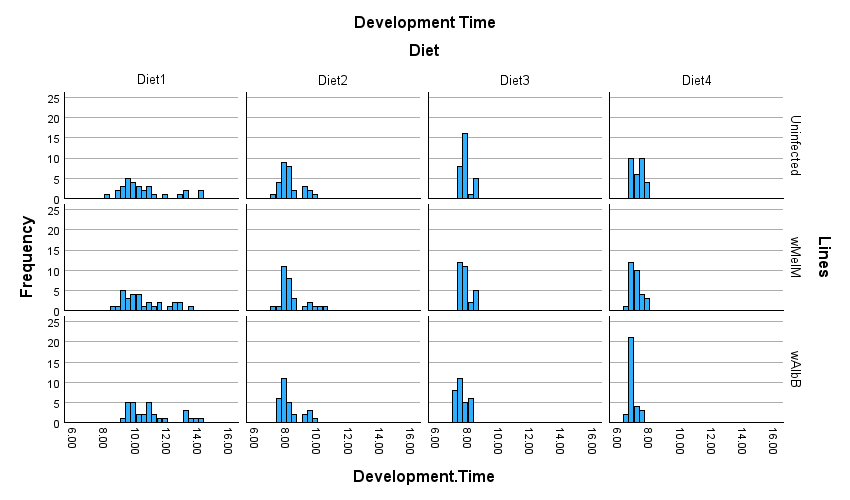


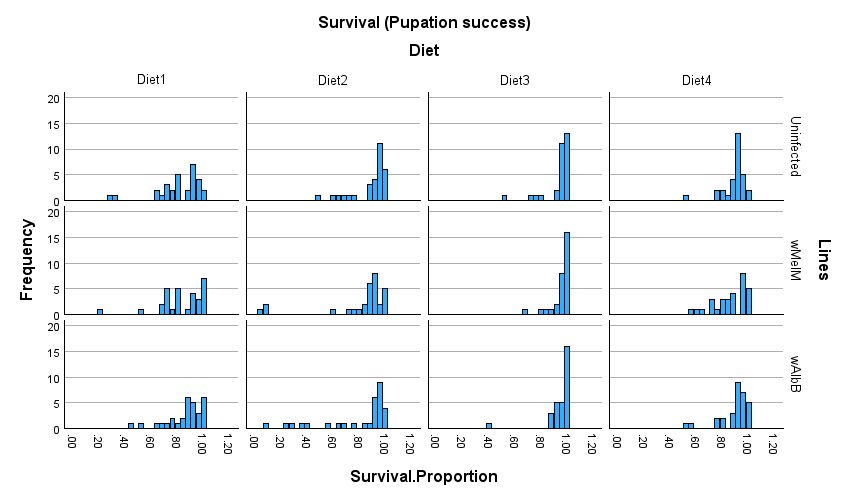


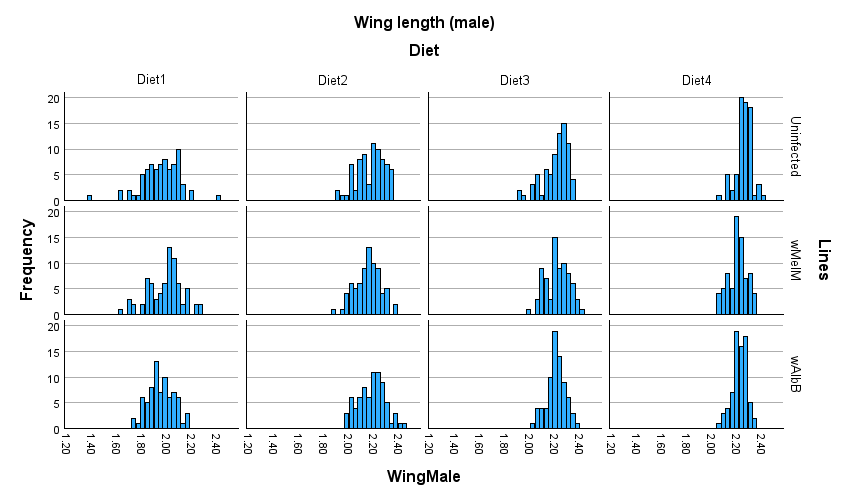


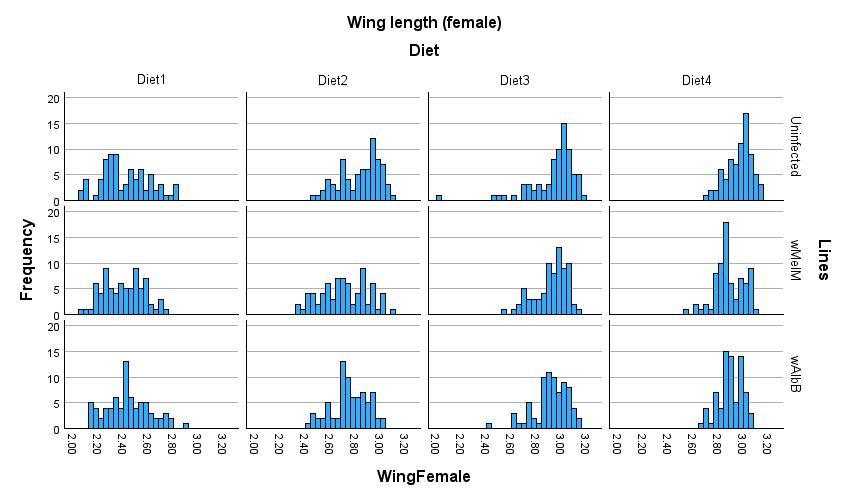


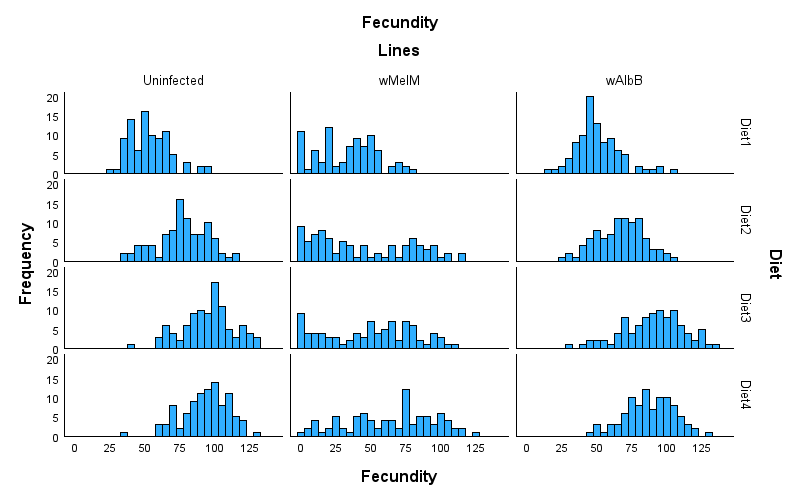


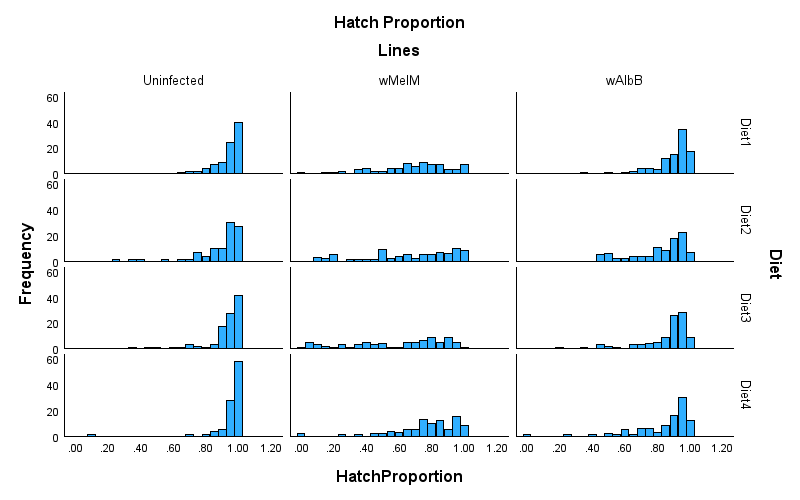

Supplement: Supplementary file 1 — Additional file 1. [file 13071_2025_6978_MOESM1_ESM.docx]
